# Femtosecond Laser-Induced Crystallization of Amorphous Silicon Thin Films under a Thin Molybdenum Layer

**DOI:** 10.1021/acsami.1c07083

**Published:** 2021-07-28

**Authors:** Nazar Farid, Adam Brunton, Phil Rumsby, Scott Monaghan, Ray Duffy, Paul Hurley, Mingqing Wang, Kwang-Leong Choy, Gerard M. O’Connor

**Affiliations:** † National Centre for Laser Applications (NCLA), School of Physics, National University of Ireland Galway, Galway H91 TK33, Ireland; ‡ M-Solv Ltd., Kidlington, Oxford OX5 1FP, U.K.; § Tyndall National Institute, University College Cork, Lee Maltings, Dyke Parade, Cork T12 R5CP, Ireland; ∥ School of Chemistry, 2nd Floor, Kane Building, College of SEFS, University College Cork, Cork T12 YN60, Ireland; ⊥ Institute of Materials Discovery, University College London, London WC1E 6BT, U.K.

**Keywords:** AM-LCD, TFT, OLED, nanorystallization, melt-free, ultrashort laser, polycrystallization, silicon

## Abstract

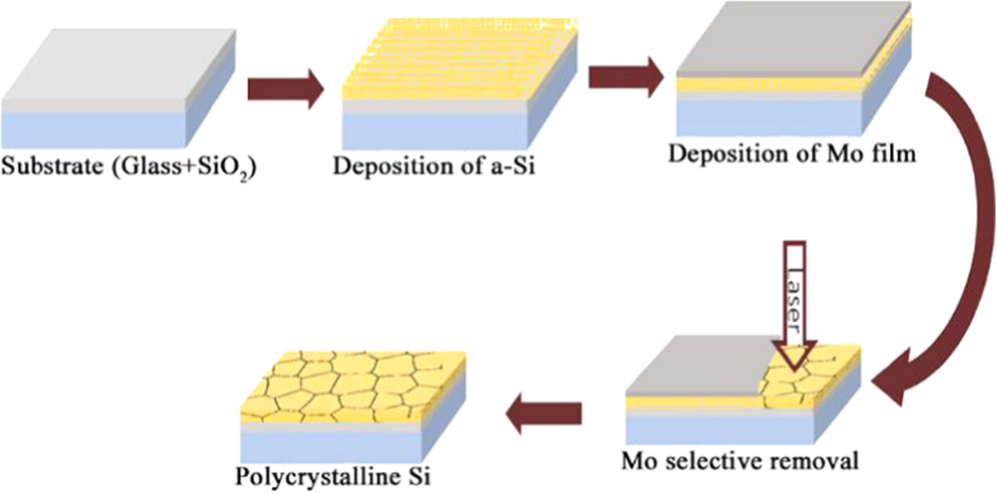

A new process to crystallize amorphous silicon without melting and the generation of excessive heating of nearby components is presented. We propose the addition of a molybdenum layer to improve the quality of the laser-induced crystallization over that achieved by direct irradiation of silicon alone. The advantages are that it allows the control of crystallite size by varying the applied fluence of a near-infrared femtosecond laser. It offers two fluence regimes for nanocrystallization and polycrystallization with small and large crystallite sizes, respectively. The high repetition rate of the compact femtosecond laser source enables high-quality crystallization over large areas. In this proposed method, a multilayer structure is irradiated with a single femtosecond laser pulse. The multilayer structure includes a substrate, a target amorphous Si layer coated with an additional molybdenum thin film. The Si layer is crystallized by irradiating the Mo layer at different fluence regimes. The transfer of energy from the irradiated Mo layer to the Si film causes the crystallization of amorphous Si at low temperatures (∼700 K). Numerical simulations were carried out to estimate the electron and lattice temperatures for different fluence regimes using a two-temperature model. The roles of direct phonon transport and inelastic electron scattering at the Mo–Si interface were considered in the transfer of energy from the Mo to the Si film. The simulations confirm the experimental evidence that amorphous Si was crystallized in an all-solid-state process at temperatures lower than the melting point of Si, which is consistent with the results from transmission electron microscopy (TEM) and Raman. The formation of crystallized Si with controlled crystallite size after laser treatment can lead to longer mean free paths for carriers and increased electrical conductivity.

## 1 Introduction

Amorphous silicon (a-Si) has highly desired optoelectronic properties like strong absorption in the visible part of the electromagnetic spectrum due to its electronic band gap of 1.8 eV.^1^ It is easy to dope a-Si with both p-type and n-type impurities. Low-temperature deposition makes it possible to prepare a-Si on thin-film devices.^2^ It is widely used for thin-film transistors (TFTs) in active-matrix liquid crystal displays (AM-LCDs),^3^ photovoltaic solar cells,^4^ solid-state photosensors,^5^ photoreceptors,^6^ and data storage devices.^7^ Even though the mobility of a-Si (0.5–1 cm^2^ V^–1^ s^–1^)^8^ is not high, it is sufficient to run 90 in. AM-LCDs up to 120 Hz frame rate as this only requires a mobility of ∼0.5 cm^2^ V^–1^ s^–1^.^9^ Higher mobilities are, however, required to run AM-LCDs at higher frame rates; this is most pertinent for organic light-emitting diode (OLED) devices as these are emissive displays where each pixel emits light proportionally to the biasing current; a-Si is not currently capable of supporting high currents due to its low mobility.

A recently developed metal oxide semiconductor, indium gallium zinc oxide (IGZO), offers higher carrier mobility (10 cm^2^ V^–1^ s^–1^).^10,11^ IGZO can be processed at low temperatures with electronic mobility higher than a-Si, which is useful for the device fabrication on heat-sensitive substrates. However, single-crystal IGZO growth with mobility of 80 cm^2^ V^–1^ s^–1^ is reported by thermal annealing of IGZO at a temperature of 700–1000 °C.^12^ The annealing temperature of crystallization may differ depending on the film preparation technique, but on average, amorphous IGZO deposited at room temperature requires thermal annealing at 600–700 °C to convert it to a polycrystalline phase.^13^ Another important issue is the thermal conductivity of IGZO (1.4 W m^–1^ K^–1^),^14^ which is significantly lower than the polycrystalline silicon (32 W m^–1^ K^–1^);^15^ this can cause self-induced thermal degradation of IGZO devices.

Polycrystalline silicon thin-film transistors (poly-Si TFTs) offer higher carrier (electron and hole) mobility (100 cm^2^ V^–1^ s^–1^)^16^ compared to a-Si and IGZO; poly-Si also has better response times and excellent stability. These properties enabled TFT dimensions to be reduced to allow a higher aperture ratio in devices resulting in increased brightness and reduced power consumption. Polycrystalline Si is a most promising material for next-generation, high-resolution ultrahigh definition (8k UHD) and high frame rate (240 Hz) display technology.^17^ There are two main routes to prepare polycrystalline Si (poly-Si) TFTs: direct deposition in a polycrystalline phase (as deposited) and phase transformation from an amorphous to poly-Si phase (crystallization process). Different approaches have been used for crystallization of a-Si, like conventional furnace annealing,^18^ rapid thermal annealing,^19^ metal-induced crystallization,^20^ field-aided lateral crystallization (FALC),^21^ and excimer laser annealing (ELA).^22^ ELA is arguably the most applied industrial process. The main advantages of ELA are crystallization from the melt phase resulting in better quality large grains. ELA is also conducted in a highly controlled environment in vacuum at elevated temperatures. The thermal budget required to crystallize a-Si is an emerging concern for heat-sensitive substrates used in new flexible technologies such as foldable smartphones and wearable devices. The challenge of scaling ELA systems for large substrate sizes is also a concern for large-screen displays such as in generation 6 or higher.^16^ High processing costs, pulse to pulse instability associated with gas lasers, and other spatial beam inhomogeneities in the crystallized region due to multipass laser scanning over large areas can present challenges in the application of ELA for TFT manufacture. Optoelectronic issues include high leakage currents and performance instabilities due to small fluctuations in current or voltage applied to the TFT devices, which can degrade the uniform brightness of OLED pixels. For example, a ±0.1 V variation in threshold voltage can change the OLED brightness by 16%.

As OLEDs are current-driven devices, the higher the current supplied, the higher the brightness. Higher mobility polycrystalline silicon can provide higher current and faster response times compared to a-Si and IGZO for the same device sizes. In OLED display devices, a pixel contains the space for both the TFT and OLED. Hence, the smaller the TFT, the larger is the space available for OLED. For high resolution, a smaller pixel size is required, and if the TFTs remain of the same size, the space for the OLED must get smaller, thus leading to a decrease in brightness. This brightness can be increased by a larger current supply, which will ultimately decrease the lifetime of the device.^8^ However, brightness can be increased by reducing the TFT size and saving more space for OLEDs. It is reported that polysilicon TFTs could provide aperture ratios of 28 and 5% larger than a-Si and IGZO, respectively.^23^

In this study, we propose a crystallization process based on a single femtosecond (fs) laser pulse to crystallize a-Si in a localized specific region on a device using solid-state diffusion without melting or generating excessive heat, which would impact nearby components. Two types of materials were used for Si selective crystallization: a commercially available a-Si-based thin-film transistor (TFT) panel and a-Si deposited on the glass substrate. The Si layer is crystallized by irradiating the upper molybdenum (Mo) layer. Mo was chosen because it has a high melting temperature. Mo has a body-centered cubic structure, whereas silicon has a diamond/face-centered cubic structure. We expected this to limit mixing. Ultrashort irradiation of electrons in Mo does not lead to significant ballistic transport of electrons from Mo to a-Si layers. We chose 40 nm thickness to ensure that most of the laser absorption took place in the Mo layer rather than the a-Si layer.

## 2 Materials and Methods

**Figure 1:**
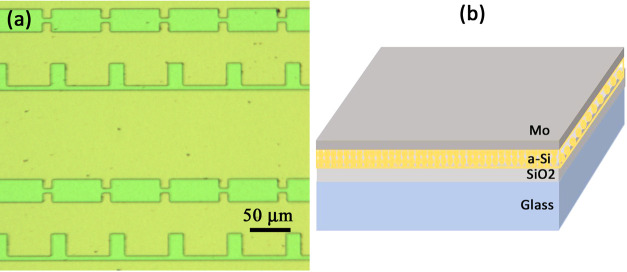
(a) a-Si-based commercial TFT and (b) a-Si deposited on the SiO_2_ dielectric layer on the glass substrate. An additional Mo layer of 40 nm thickness was deposited on a-Si prior to crystallization.

Two types of materials were used for selective crystallization of a-Si: a commercially available amorphous silicon (a-Si)-based thin-film transistor (TFT) panel and a-Si deposited on the glass substrate (Figure 1). In the purposely fabricated samples, a dielectric SiO_2_ film of 216 nm thickness was deposited on the glass substrate (0.5 mm thick), and subsequently, a 40 nm thick a-Si film was then deposited using a chemical vapor deposition (CVD) technique. Prior to laser irradiation, both types of samples were coated with an additional 40 nm thick molybdenum (Mo) layer by a magnetron sputtering technique.

A femtosecond laser (amplitude S-pulse) delivering 500 fs pulse duration at the wavelength of 1030 nm that can operate at a single pulse to 300 kHz was used for crystallization. The samples were placed on a three-dimensional (3D) computer-controlled stage (Aerotech Inc.), which enabled the sample position to be changed with sub-micrometer accuracy. The laser was focused on the sample with 10 cm focal length lens (NA = 0.014) in the scanning system, which is coupled to the machining stage through a combination of different reflectors and mirrors. The galvanometer-based beam scanning system (SCANLAB) is used to direct the beam over the sample surface by adjusting the speed of the steering mirrors. The laser was operated at a maximum power and attenuated using a combination of half-wave plate and polarizer to keep the optimal beam shape and to get highest pulse to pulse stability. The degree of crystallinity of Si was evaluated in situ by Raman spectroscopy using an Ar-ion laser of a wavelength of 514.5 nm as the excitation source. The focused green laser beam has a diameter of ∼3 μm, and the penetration depth (d) can be estimated by the following relation^24^
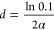
1where α is the absorption coefficient of Si at the wavelength (514.5 nm) of the incident laser. The penetration depth is estimated to be 760 nm for 514 nm laser, which is quite larger than our Si film thickness (40 nm). Surface morphology was characterized using a scanning electron microscope (SEM). High-resolution transmission electron microscopy (TEM) was used to characterize the crystallization and electron energy loss spectroscopy (EELS) was used for elemental distribution at the interface to rule out the diffusion of Mo at the interface. Electrical resistivity was measured using a four-point probe method, while the improvement in the electron mobility after crystallization was attempted using the AC and DC Hall Effect methods.

## 3 Results and Discussion

**Figure 2:**
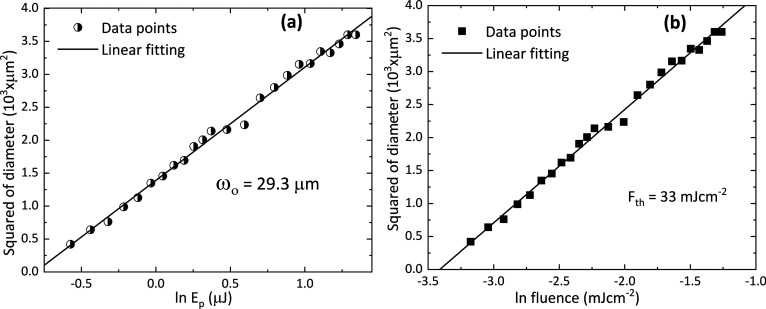
Semilogarithmic plot between applied pulse energy and squared of crater diameter (a) and between the log of fluence and squared of diameter (b).

As the laser had a Gaussian beam shape, the spot diameter (2ω_o_) at 1/e^2^ was calculated experimentally using Liu’s method as provided by the following relation^25^
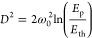
2where *D* is the diameter of crater, ω_o_ is the radius at the waist of the Gaussian-shaped beam at the focus, *E*_th_ is the threshold energy, and *E*_p_ is the pulse energy. The beam radius is obtained from the slope of the plot between logarithmic ln *E*_p_ against *D*^2^, as shown in Figure 2a. ω_o_ at the focus was estimated to be 29.3 ± 0.12 μm. After single pulse irradiation, the Mo film and debris were removed using an adhesive sticky tape. It should be noted that the Mo film was not possible to be removed at a region where the laser pulse was not incident or where the fluence was particularly low. The fluence is calculated by the equation in Liu’s relation;^25^*F* = *2E*_p_/πω_0_^2^ and the ablation threshold (*F*_th_) is defined as the fluence value at which the Mo film starts to peel from the Si surface using an adhesive tape.

**Figure 3:**
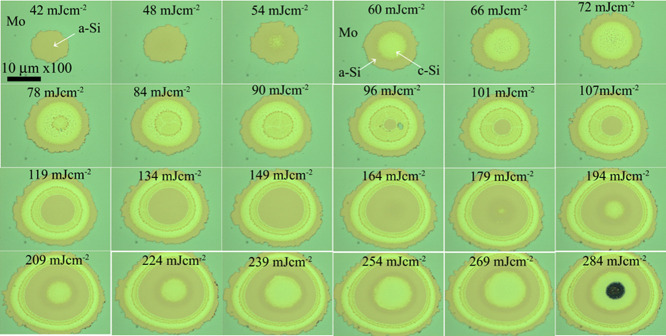
Mo thin film removal by a single fs laser pulse and crystallization of the underlying a-Si thin film at different laser fluences.

A fluence of 33 mJ cm^–2^ was defined as the Mo removal threshold because at this fluence the Mo film started to peel off using the sticky tape. Mo is itself directly ablated from the Si surface at a fluence of 75 mJ cm^–2^. Figure 3 shows the crater formation as a result of Mo removal from a-Si surface with single pulse irradiation at different laser fluences. The Mo thin film undergoes cracking at a low fluence but remains intact with the Si surface and could be removed easily with the sticky tape. While the Mo film starts to be removed with the sticky tape from 33 mJ cm^–2^, there is no crystallization of the underlying a-Si surface until 54 mJ cm^–2^. Crystallization begins from 54 mJ cm^–2^.

Once the incident laser energy increased beyond the removal threshold (33 mJ cm^–2^), Mo thin film starts to delaminate from the center of the laser crater without affecting the underlying a-Si surface. a-Si starts to crystallize at 54 mJ cm^–2^ from the center of the crater with increasing the applied fluence. The crystallized region increases with the applied laser fluence up to a certain fluence range. The Si crystallization process based on the laser fluence can be divided into three key regimes.

### 3.1 Regime I (Low-Fluence Regime: 54–90 mJ cm^–2^)

At a low fluence, above the ablation threshold (33 mJ cm^–2^), Si crystallization starts at a fluence of ∼54 mJ cm^–2^ from the center of the laser crater. The range of Regime I starts from 54 mJ cm^–2^ and extends up to 90 mJ cm^–2^.

**Figure 4:**
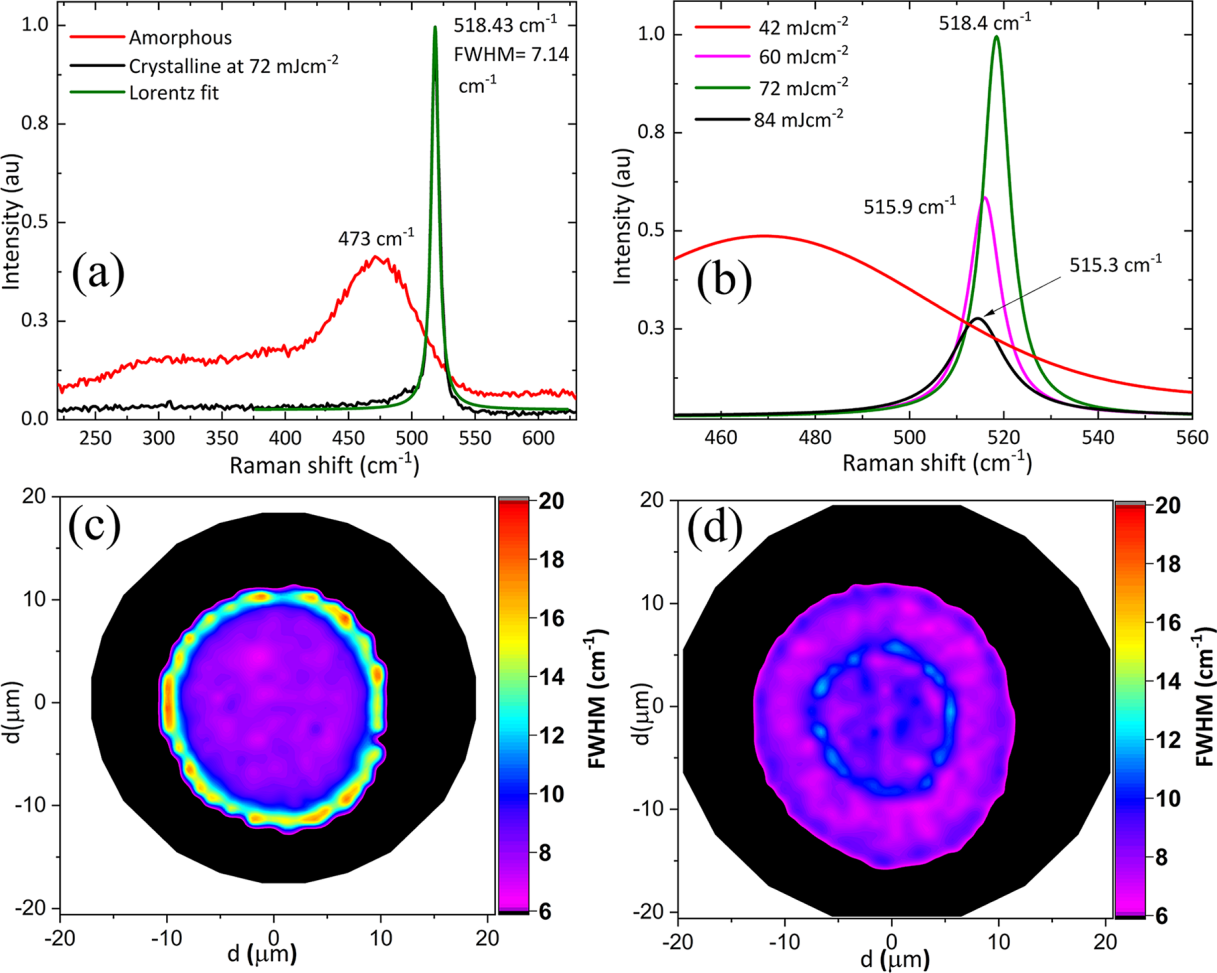
(a) Raman spectra of a-Si and laser-induced crystallized Si at 72 mJ cm^–2^. Raman peak at 473 cm^–1^ corresponding to amorphous and the peak at 518.43 cm^–1^ corresponding to a crystalline phase. (b) Variation in width and position of Raman peak, corresponding to the Si crystalline phase, with fluence. The Raman maps of the laser-crystallized area with a single pulse at 72 mJ cm^–2^ (c) and 90 mJ cm^–2^ (d) generated using the spectral line width of the peak corresponding to the crystalline phase.

Figure 4a shows the Raman spectra of a-Si and the generated nanocrystalline silicon (nc-Si) after single pulse irradiation at 72 mJ cm^–2^. The Raman spectrum of a-Si consisting of a broad peak at 473 cm^–1^ corresponding to scattering by optical phonon modes and the peak at 518 cm^–1^ is due to a crystalline phase.^26^ The peak parameters (position, amplitude, and the width) related to the Raman scattering were determined by fitting Lorentzian function. If the Si crystallite dimension is less than 50 nm, then the Raman peak for nc-Si is positioned between 500 and 520 cm^–1^ depending on the dimensions of the grains.^27^ Hence, crystallized Si should be in the form of nanocrystallites. Figure 4c shows the Raman map of the phonon width for a region crystallized with a single pulse at 72 mJ cm^–2^. As the phonon lifetime decreases due to the defects acting as anharmonic perturbations, the distribution of crystallite dimensions causes the broadening of the Raman peak. Therefore, the map was generated using the full width at half-maximum (FWHM) of the crystallized Si Raman peak after Lorentzian fitting. The map shows that the current process offers a high spatial control over the quality of crystallization. The central part of the spot is particularly uniformly crystallized as there is no significant difference in FWHM and in the corresponding Raman peak position. The central crystallized region is increased with fluence in the range from 54 to 90 mJ cm^–2^. From a fluence of 78 mJ cm^–2^, a second region, surrounded by a circular ring, appeared at the center of the spot and the diameter of this region increased with increasing fluence, as shown in Figure 3. Figure 4b shows how the spectral shift and width of Raman peak from crystallized silicon from the center of spot varies with fluence. It is evident from the Raman analysis that the crystallite size changes with fluence as the spectral shift is different at different fluences. At fluences of 78, 84, and 90 mJ cm^–2^, two different crystallized regions can be seen in Figure 3. To establish the uniformity of these crystallized regions, a Raman mapping performed for Si spot crystallized at 90 mJ cm^–2^ is given in Figure 4d. No significant difference in the line width was observed; however, the Raman peak was little broader at the interface between the inner and out annular regions formed by this ring feature.

### 3.2 Regime II (No Crystallization: 96–149 mJ cm^–2^)

Interestingly, from a fluence of 96 mJ cm^–2^, the crystallization of Si at the center of the spot stopped and the surface of a-Si appeared as unaffected. However, the periphery of the crater was still crystallized in the form of a ring. The central unaffected a-Si region extended with increasing fluence values up to 150 mJ cm^–2^. This effect is attributed to the physical contact between the Mo and Si layer. At low fluence, the electrical contact is maintained throughout the laser interaction. At high fluence, sufficient energy is exchanged from the Mo to the Si layer prior to delamination. In Regime II, insufficient energy is exchanged for crystallization to occur at the center prior to delamination.

### 3.3 Regime-III (High-Fluence Regime Crystallization: 164–269 mJ cm^–2^)

**Figure 5:**
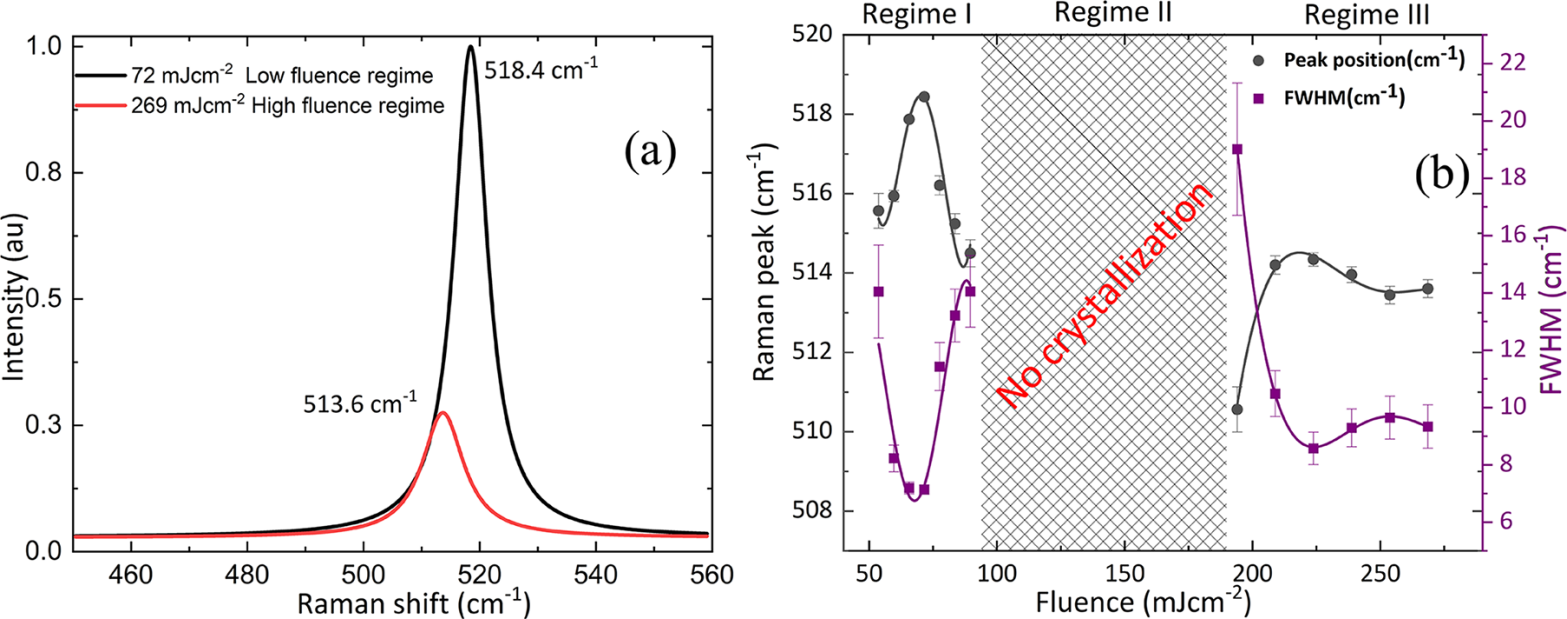
(a) Raman spectra of laser-crystallized Si at low-fluence regime (72 mJ cm^–2^) and higher-fluence regime (269 mJ cm^–2^). (b) Fluence regimes for Si crystallization variation in a position of Raman peak for the crystallized phase of Si and its width with fluence.

At the center of the laser spot, Si starts to crystalize again from the fluence of 164 mJ cm^–2^ as confirmed from Raman spectrum shown in Figure 5. At higher fluences, we propose that sufficient energy is transferred from the Mo layer to a-Si prior to delamination. However, for a perfect crystalline phase, the amorphous Raman peak, which is centered at ∼473 cm^–1^, should be zero at 164 mJ cm^–2^; however, it is not. This indicates that a mixed phase of amorphous and crystalline regions coexist. To estimate the degree of crystallinity of Si in the mixed crystalline and amorphous phase, the Raman signal is decomposed into two Gaussian peaks centered at 517 cm^–1^ (crystalline phase) and 473 cm^–1^ (amorphous phase contribution), using the method as provided in ref (28)
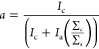
3where *I*_c_ and *I*_a_ are the integrated intensities of peaks correspond to crystalline and amorphous phases, respectively, and ∑_c_ and ∑_a_ are the integrated Raman cross-section of the optical phonon modes for crystalline and amorphous Si, respectively. The value of the ratio (∑_c_/∑_a_) is almost 1 for nanocrystallites and then exponentially decreases for larger crystallites. The central part of the crater starts to crystallize completely at 179 mJ cm^–2^, and the crystallized region extends with fluence until 269 mJ cm^–2^ when the Si layer damages with increasing fluence, as shown in Figure 3.

It is predicted from the Raman analysis that the size of crystallites is smaller than that crystallized in a low-fluence regime, as shown in Figure 5a. Figure 5b summarizes the Raman analysis of crystallized Si at different fluences.

**Figure 6:**
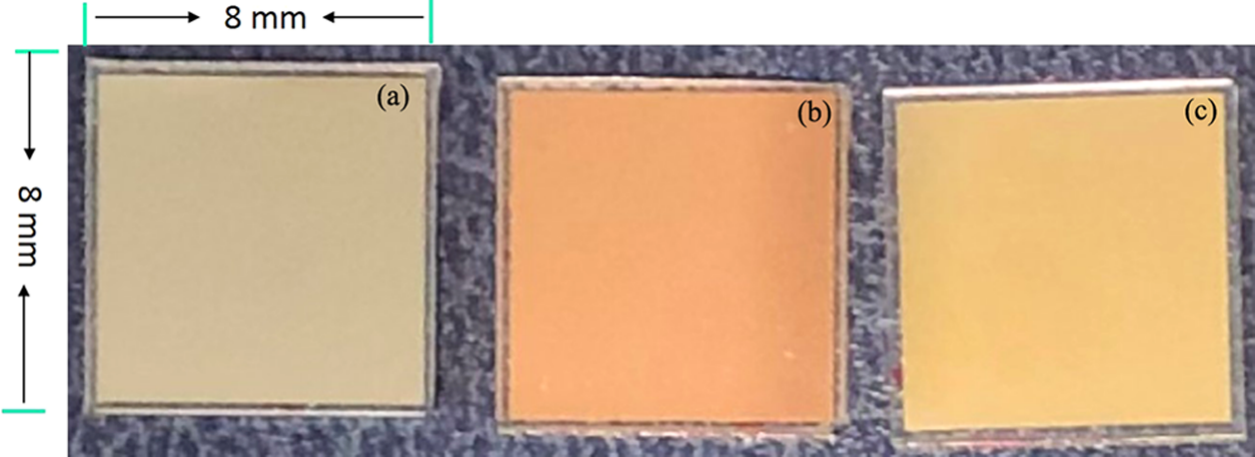
(a) a-Si with Mo on top, (b) a-Si, and (c) crystallized Si after laser crystallization.

To explore the potential of the process for a large area crystallization, we demonstrated the crystallization on a large area using an industrial laser scanning system. Figure 6a shows the optical microscope image of a-Si with Mo coating on top, while Figure 6b,c presents images of a-Si and crystallized Si, respectively. A yellowish-brown color of a-Si film was changed to a light yellow color after crystallization; these color changes are indicative of the surface reflectance, which results from the phase changes of the materials upon crystallization.

The undoped a-Si samples presented in Figure 6 were subjected to electrical characterization. The resistivity before and after laser crystallization reduced from 280 to 80 kΩ m^–1^. It is proposed that this is a very reasonable improvement for undoped a-Si. Attempts to determine the free carrier mobility using a Hall technique had an insufficient signal-to-noise discrimination to be reliable due to the high resistance of undoped a-Si.

### 3.4 Direct Laser Crystallization of a-Si

**Figure 7:**
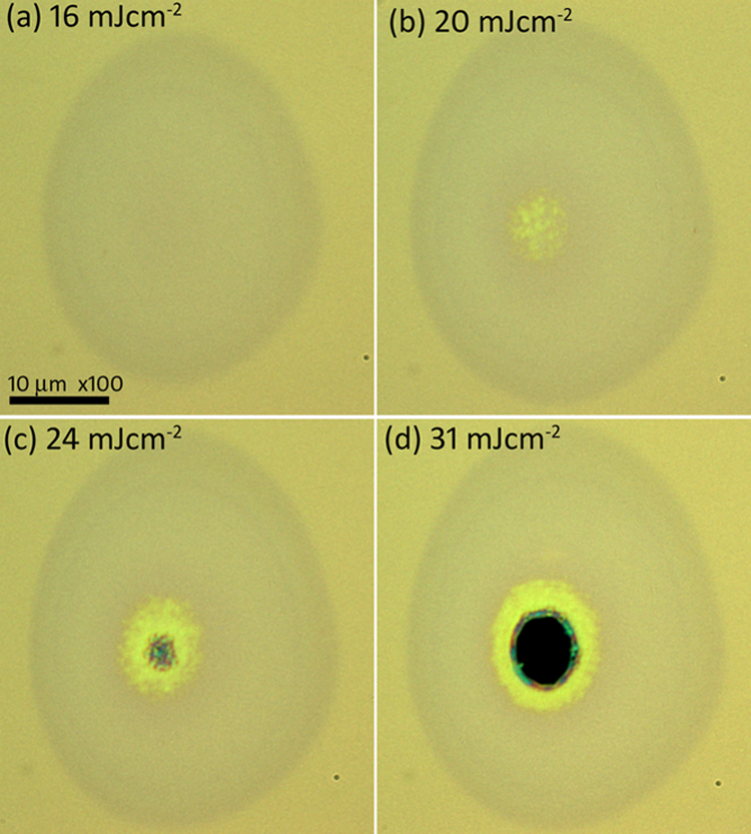
Laser-generated craters produced with a single laser pulse on the a-Si thin film without the presence of a Mo additional film at laser fluences of (a) 16 mJ cm^–2^, (b) 20 mJ cm^–2^, (c) 24 mJ cm^–2^, and (d) 31 mJ cm^–2^. A mixed phase, partially crystallized Si region, is observed at a fluence of 20 mJ cm^–2^. At fluence of 24 mJ cm^–2^, the a-Si thin film starts to damage from the center of the beam and is defined as a damage threshold.

To check the figure of merit presented by the crystallization process with the additional Mo layer, a-Si thin film was directly irradiated with the same femtosecond laser. Figure 7 shows the modified spots on the a-Si thin film after irradiation with a single pulse at different laser fluences. From a fluence of 16 mJ cm^–2^, the color of the thin film starts to change on the irradiated laser spot, as shown in Figure 7a, but the a-Si phase was not changed. The central region of the irradiated spot, where the laser energy was higher because of the Gaussian nature of beam starts to be crystallized at a fluence of 20 mJ cm^–2^ (Figure 7b). On increasing the fluence a little higher, the crystallized region increased, but the central part of the film starts to damage with partial ablation occurring, as shown in Figure 7c. The yellow region at the center of spot is crystallized with mixed phases. A fluence value of 24 mJ cm^–2^ at which the a-Si starts to be damaged is defined as the damage threshold fluence. The a-Si film is completely damaged at a fluence higher than the damage threshold, as evident from Figure 7d. In comparison to this direct laser-induced crystallization, we propose that the crystallization of Mo layer provides a greater process window for laser-induced crystallization of a-Si.

**Figure 8:**
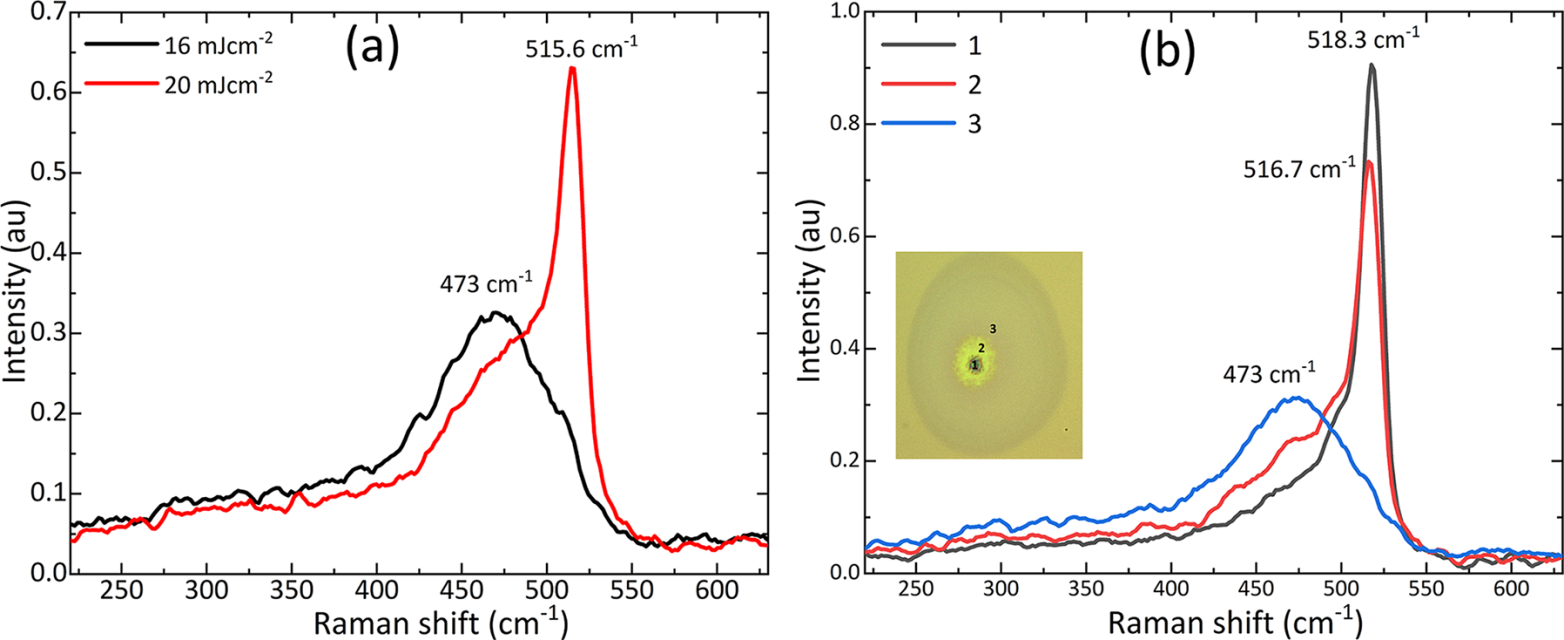
(a) Raman spectra obtained from the center of the laser spot at fluences of 16 and 20 mJ cm^–2^ on a-Si thin film without a Mo layer (b) Raman spectra at different positions on the across laser spot at 24 mJ cm^–2^ on a-Si thin film without the additional Mo layer.

Figure 8a shows the Raman spectra from the center of laser spots on the a-Si thin film after irradiation with a single pulse at fluences of 16 and 20 mJ cm^–2^. No crystallization and structural changes were observed at a fluence of 16 mJ cm^–2^, while a-Si was crystallized at a fluence of 20 mJ cm^–2^ with a mixed crystallized and amorphous phase as confirmed by the Raman analysis. This composite crystallized and amorphous phase is consistent with the previously reported results.^29^ The degree of crystallization increased with increasing fluence, but partial ablation/damage starts from the center, as denoted by the black region, as shown in Figure 7c. Raman measurements were performed at different locations within the laser spot generated at 24 mJ cm^–2^, as shown in Figure 8b. As the Raman probe laser is moved from the center of the laser spot, the degree of crystallinity of Si is decreased, and at point 3, it is entirely amorphous, as shown in Figure 8b. This is in contrast to the previous study where a femtosecond laser was reported to create inhomogeneous crystallization within the laser created spot at a wavelength of 800 nm and at a fluence range of 377–613 mJ cm^–2^.^30^ In that study, no crystallization was observed at the center of irradiated spot while there was increasing crystallization extending from the center toward the edge of the irradiated spot. This result contradicts with the results found in our study, and this could be due to the different wavelength, pulse duration, and higher value of fluence used in the previously reported study.

The mechanism of direct laser crystallization of a-Si is different from the present process, where a-Si is not directly irradiated with the laser, but where the energy is absorbed by the Mo film and then transferred to the underlying Si film. In direct ultrashort laser pulse semiconductor interactions, the laser energy is transferred first to the electrons and then to the lattice. After laser irradiation, a semiconductor undergoes several stages before returning to an equilibrium-like carrier excitation, carrier thermalization, carrier removal, and thermal and structural changes of the lattice.^31^ If the photon energy is greater than the band gap, then single-photon absorption is the dominant mechanism for excitation of valence band electrons across the band gap to the conduction band. However, as the band gap of silicon is greater than the laser photon energy (1.2 eV), the multiphoton absorption is the main excitation mechanism. For indirect band gap semiconductors, as in silicon, single-photon absorption can be dominated if the photon’s energy is greater than the indirect energy gap, but phonon assistance is necessary to conserve momentum. If there are free carriers in the semiconductor, then free carrier absorption increases the energy of carriers in the electron and hole plasma. The number density of free carriers does not change with absorption, but the energy of the free carrier gas increases. If the laser photon energy is high enough to cause the excitation of free carrier above the band gap (for semiconductor) or above the Fermi level (in case of metal), impact ionization can generate additional excited carriers.^31,32^ Thus, the absorption process in semiconductors is complex. Covalent bonding holds the semiconductor structure together, and it is stable only, if electrons are in the ground state. Covalent bonding breaks down on the excitation of electrons from stable valence band state to antibonding conduction states. During ultrashort pulse excitation, if enough electrons populate excited antibonding states in the conduction band, this causes the lattice structure to become unstable, and as a result, bond breaking occurs. It has been reported that if enough bonds are broken, roughly 10% of the valence electrons, then the lattice will begin to deform as certain phonon modes become soft.^32^ If the excited electrons reach a critical density (10^22^ cm^–3^), the structure will become unstable, and a nonthermal phase transition can occur, if 10–15% bonds are broken in the lattice.^32,33^ Hence, ultrashort laser pulses can induce a lattice instability by generating a dense photoexcited plasma, which can weaken the lattice that promotes the ionic movement over a significant fraction of the bond length without significantly increasing their thermal energy.^34^ Therefore, the weakening of lattice by high-density electron and hole plasma could be the main mechanism for the nonthermal phase change in direct femtosecond laser annealing of silicon at low fluence.^35^ The laser molybdenum silicon interaction is described in Section 5.

### 3.5 Structural Analysis

**Figure 9:**
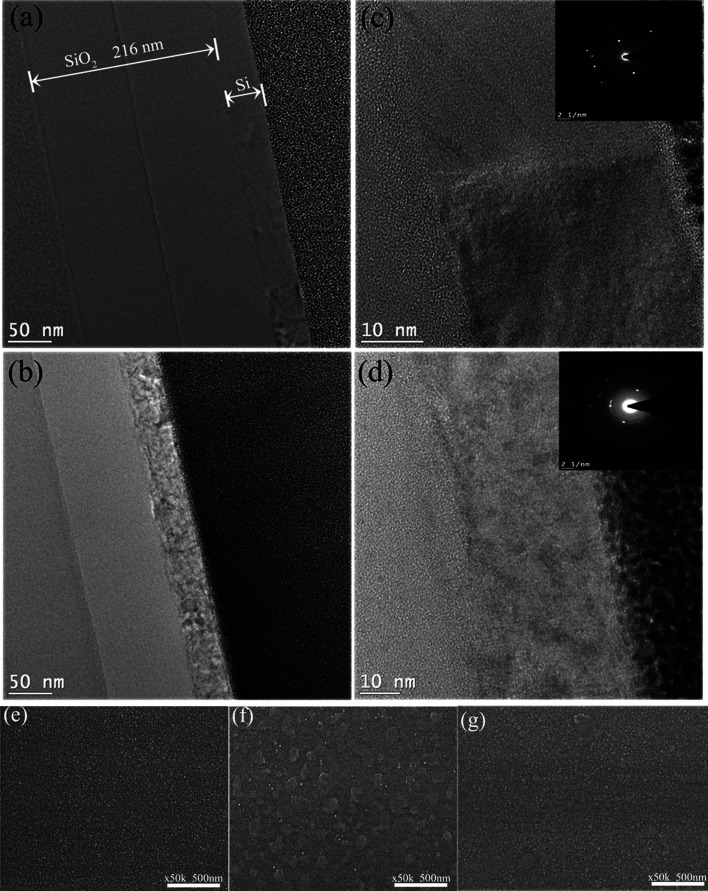
(a, b) TEM cross-section images of samples after crystallization at 72 mJ cm^–2^ (Regime I) and at 269 mJ cm^–2^ (Regime III), respectively. (c, d) Higher-magnification images of (a) and (b), respectively, while insets are their respective diffraction patterns. (e) High-resolution SEM image of a-Si surface before crystallization and SEM image from the center of crater after the crystallization at (f) 72 mJ cm^–2^ and (g) 269 mJ cm^–2^.

One of the issues that pertain to the Mo layer could be the diffusion of Mo into Si. During the thermal annealing of Mo–Si systems, silicide formation has been observed on annealing at 325 °C for several hours; the diffusion of Mo into Si was also observed when the time was reduced to less than a minute on heating at 600 °C.^36−38^ In the case of fs laser pulses, silicide formation can only take place if Mo or Si atoms diffuse across the interface. The atomic diffusivity (*D*) through the interfaces of Mo–Si layer is estimated to be (4 ± 2) × 10^–4^ nm^2^ s^–1^ at 530 °C;^39,40^ using an Arrhenius’s dependence, it can be estimated that the diffusion coefficient is ∼1.5 × 10^–3^ nm^2^ s^–1^ at a temperature just below the melting temperature of silicon.^41^ Assuming heat conduction from Mo to Si film in a time shorter than 1 μs, the diffusion length can be calculated to be (4*D* × *t*)^1/2^ = 7.7 × 10^–5^ nm, which is significantly smaller than the size of atoms. Atomic diffusion and resulting silicide formation at the interface can thus be neglected below the melting temperature of amorphous silicon at this relatively short time scale.^39^ To investigate the Mo/Si diffusion and silicide formation at the Mo–Si interface, high-resolution EDX and EELS coupled with TEM was performed. No evidence of mixing or silicide formation at the interface was found. Figure 9a shows the cross-section of a sample after performing laser crystallization. The 40 nm Si layer can be clearly seen on the SiO_2_ dielectric layer of thickness 216 nm deposited on the glass substrate. The crystallites with different sizes are shown with different contrast in the TEM dark-field images exhibiting the different contrast. In Regime I, Si was crystallized with crystallites sizes from 10 to 40 nm. Figure 9c shows the magnified image of Figure 9a, while the inset shows the diffraction pattern of well-crystallized silicon. In contrast to low-fluence Regime I, small crystallites with sizes ranging from 3 to 5 nm were generated in Regime III, as given in Figure 9b. Figure 9d shows the high magnification, while the inset is a diffraction pattern of less well-crystallized silicon.

Figure 9e–g shows the SEM images of the surface structure of the Si film before (e) and after crystallization at fluences of 72 mJ cm^–2^ (f) and 90 mJ cm^–2^ (g). Small particles are observed in the a-Si film, while particles grow larger at 72 mJ cm^–2^ (low-fluence regime) as compared to 90 mJ cm^–2^ (higher-fluence regime). TEM and SEM analyses confirm vertical and lateral grain growth and crystallization. TEM and SEM results are consistent with the Raman results.

## 4 Numerical Simulation

In fs laser–metal interactions, the laser energy absorption and resulting heating, melting, or ablation can be explained by a two-temperature model (TTM)^42^
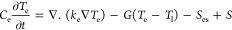
4
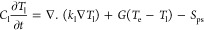
5
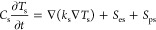
6where *T*_e_(*T*_l_), *C*_e_(*C*_l_), and *k*_e_(*k*_l_) are electron(lattice) temperature, electron(lattice) heat capacity, and electron(lattice) thermal conductivity of Mo, respectively. *G*(*T*_e_) is the temperature-dependent electron–phonon coupling coefficient of Mo, and *S*_es(ps)_ = *h*_ep(pp)_/*d*(*T*_e(l)_ – *T*_s_) represents the heat exchange from Mo to Si at the interface because of thermal boundary conductance for electrons (*h*_ep_) and phonons (*h*_pp_). *T*_s_, *C*_s_, and *k*_s_ are the lattice temperature, heat capacity, and thermal conductivity of Si, respectively. *S* is the temporal and volumetric energy from the laser source of Gaussian profile which can be defined as
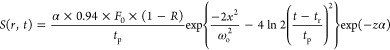
7where *F*_0_ is laser fluence, *R* is reflectivity of Mo, *t*_p_ is laser pulse duration, *ω*_o_ is the radius of the beam waist, *t* is time, and *t*_r_ is reference time. The thermodynamical and optical properties of Mo and Si used in the simulations are listed in Table 1.

**Table 1:** Thermodynamical and Optical Properties of Mo and Si Used in Modeling

			Mo	Si	SiO_2_
melting point	*T*_m_	K	2896^49^	1420^50^	1973
heat capacity	*C*_l_	J m^–3^ K^–1^	2.56 × 10^6^ @ 300 K^51^	3.14 × 10^6 ^^52^	2.27 × 10^6^
				1.6 × 10^6 ^^53^	
				2.3 × 10^6 ^^54^	
thermal expansion coefficient	α_exp_	K^–1^	4.9 × 10^–6^ @ 400 K^55^		4.9 × 10^–7^
electron–phonon coupling constant	*G*	W m^3^ K^–1^	4.8 × 10^17^ @ 300 K	1.36 × 10^18 ^^56^	
thermal heat conductivity	*k*	W m^–1^ K^–1^	138 @ 300 K^57^	∼0.9,^58^ 1.7^59^	1.1–1.4
				4.1^60^	
				0.94^61^	0.81^62^
				0.48^50^	
electron thermal conductivity	*k*_e_	W m^–1^ K^–1^	105 @ 300 K^63^		
lattice thermal conductivity	*k*_l_	W m^–1^ K^–1^	33 @ 300 K^63^	124^63^	
reflectance	*R*		0.671^64^		
absorption coefficient	α	m^–1^	5.1 × 10^7^ @ 1033 nm ^65^	7.9 × 10^4 ^^66^	
Fermi velocity	*v*_F_	ms^–1^	9.18 × 10^5^^67^		
			1.7 × 10^6^^68^		
thermal diffusivity	*k*_f_	m^2^ s^–1^	5.4 × 10^–5^		
Debye temperature	θ_D_	K	430 ± 20^51^	487^52^	
specific heat constants	γ	J m^–3^ K^–2^	198^51^		
mass density	ρ	kg m^–3^	10 280^69^	2330^54^	2270
longitudinal velocity	*v*_l_	ms^–1^	6.25 × 10^3^^53^		
transverse velocity	*v*_t_	ms^–1^	3.35 × 10^3^^53^		
interaction constant	β_l_		10^53^		
	β_t_		35^53^		
electron relaxation time	τ_e_	s	3.5 × 10^–15 ^^68^		

Energy transfer across the interface in a metal–semiconductor thin-film system is complicated. In metals, electrons dominate in heat transfer, while phonons dominate in semiconductors. Hence, the energy exchange between Mo and Si must occur between electrons and phonons to transport heat across the Mo–Si interface. There are two possible mechanisms: direct phonon transport (*h*_pp_) and inelastic electron scattering at the Mo–Si interface (*h*_ep_). In the case of electron elastic scattering, electrons reflect elastically from the Mo–Si interface and thermalize with phonons on the Mo side. Coupling between electrons from the Mo film and phonons on the Si thin film through anharmonic interactions causes the inelastic scattering of electrons at the interface and results in energy exchange with phonons in the a-Si material. The total conductance *h*_eff_ at the Mo–Si interface^43^
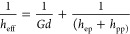
8where *h*_pp_ is phonon–phonon conductance and can be calculated using the diffuse mismatch model.^43,44^ In TTM simulation, we used 3.7 × 10^8^ W m^–2^ K^–1^ as the *h*_pp_ value that was measured at 300 K.^45^ The parameter, *h*_e_*= Gd*, is the nonequilibrium contribution to the total conductance because of thermal transfer associated with electron–phonon coupling in the Mo film; this can increase with film thickness and for metals having a large electron–phonon coupling like Mo. The corresponding resistance becomes negligible compared with the phonon–phonon  and electron–phonon interfacial resistances. The direct coupling between the Mo electrons and the Si phonons at the interface is estimated using the model proposed by Sergeev^46,47^
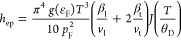
9where *g*(ε_F_) is the electron density of states at the Fermi level, *p*_F_ is the Fermi momentum, θ_D_ is Debye temperature, *T* is the temperature at the interface, and *v*_l_ and *v*_t_ are the longitudinal and transverse sound velocities, respectively. The integral term  is for higher temperatures; for lower temperature *J(y)* = 1. β_*l*_ and β_*t*_ are the interaction constants for electrons that describe the coupling of electrons with thermal longitudinal and transverse phonons, respectively. These are related to one another by^48^
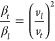
10where , *v*_o_ is the two-spin density of electron states, and ρ is the density of Mo. The values of the thermophysical parameters used in simulations are provided in Table 1.

**Figure 10:**
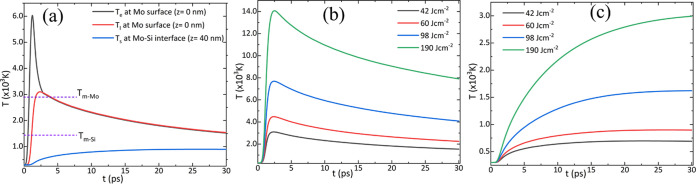
(a) Temporal evolutions of electron and lattice temperatures at a fluence of 60 mJ cm^–2^ and at the center of the irradiated spot on the Mo surface (*z* = 0) and at the Mo–Si interface (*z* = *d* = 40 nm). Simulations correspond to a fluence of 60 mJ cm^–2^ at which the Si film was crystallized. (b) Temperature evolution at the surface of Mo and (c) at the Si surface at different laser fluences. *T*_m_-Si refers to the melting temperature of a-Si.

Figure 10a shows the numerically simulated electron and lattice temperature evolution at the Mo surface with time for a fluence (60 mJ cm^–2^), where the Si film was crystallized in Regime I. After the incidence of the laser beam on the Mo surface, electrons in the Mo film get excited after laser energy absorption through photon–electron coupling. The time required for these nonthermal excited electrons to thermalize and occupy a distribution of excited states is called the electron relaxation time (τ). This characteristic relaxation time defines whether the transport of these excited nonequilibrium electrons will be diffusive or ballistic; it is typically of the order femtoseconds. The ballistic transport of electrons is favored if the relaxation time is long. Electrons re-establish a Fermi distribution in these stabilized excited states, while the lattice remains undisturbed. Such an excited electron bath is initially localized within the optical absorption depth, but later diffuses to deeper parts of the film due to electron temperature gradient. The diffusion length of electrons depends on electron-lattice coupling. After the laser is incident, the electron temperature first increases rapidly compared to lattice temperature and then reaches a peak temperature due to the relatively small heat capacity of electrons, as shown in Figure 10a. At the surface of Mo, the electron and the lattice temperatures reach thermal equilibrium just after 2.6 ps due to the high value of *G*. After the thermal equilibrium, the temperature then decreases with time due to the combined heat diffusion; the absorbed laser energy remains stored in thermalized electrons and diffuses into the deeper part of the film, while simultaneously interacting with the lattice resulting in heating of material. At this fluence, the lattice temperature at the Mo surface reaches a maximum value of 1494 K at 2.19 ps after the laser pulse was on. The underlying Si surface reaches a maximum temperature of 560 K at 22 ps after the laser pulse is incident on Mo and 19.81 ps after the peak temperature is reached in Mo.

Figure 10b,c shows the lattice temperature at the Mo surface and in Si at the Mo–Si interface with different laser fluences. The simulation predicts that Mo and Si temperatures reach a melting point at a fluence of 185 mJ cm^–2^. No crystallization of a-Si was observed at a fluence of 42 mJ cm^–2^, and at this fluence, the simulation predicts the maximum lattice temperature at Mo and Si surfaces are 1494 and 560 K, respectively. Figure 10b,c shows that temperatures in neither Mo nor Si reached the melting points for Regimes I and II. In Regime III, the simulated lattice temperature at the Mo and Si surface is higher than the melting temperature of Mo and Si. This suggests that crystallization in Regime III occurs after melting and resolidification of Si.

## 5 Laser Metal-Induced Crystallization

The study demonstrates how irradiating a thin layer of molybdenum can enhance the process window and the associated control of laser metal-induced crystallization of silicon compared with other processes based on direct exposure of silicon. The molybdenum layer absorbs the ultrashort laser pulse in a controlled way—free from the complexities associated with a semiconductor band gap. The rapid thermalization of hot electrons establishes an electron temperature in molybdenum and lessens the potential for damage by deeply penetrating ballistic electrons. Electrons in this thermalized electron gas either couple to phonons in molybdenum or diffuse and scatter from the Mo–Si interface. Scattering from the Mo–Si interface can be either elastic or inelastic. Elastic scattering at the interface results in further electron–phonon coupling in the molybdenum layer. Inelastic scattering at the interface results in electron–phonon coupling between electrons in molybdenum and phonons in silicon. In addition, energy transfer can also take place between phonons in molybdenum and phonons in silicon.

These thermal energy transfer processes are only effective when molybdenum and silicon layers are in physical contact. The contact of these layers is determined by the abruptness of the interface and the applied laser fluence. The sharpness of the interface is determined by the different body-centered and face-centered crystalline structures for Mo and Si, respectively. The rate at which the fluence is applied to the laser pulse determines when the onset of delamination of molybdenum from the silicon layers occurs. In Regime I (Figure 5), the Mo layer remains in contact long enough for sufficient thermal energy transport to occur and for solid-state crystallization to take place. In Regime II, the Mo layer is removed prior to sufficient energy transfer for crystallization to take place; hence, the region in the center of the pulse is not crystallized. In Regime III, sufficient energy transfer takes place for the onset of melting and for recrystallization to take place even though the molybdenum layer is removed by the application of the large laser fluence in the center of the laser pulse.

The above description is consistent with the findings from simulations which predicts that Si crystallization in Region I occurred at a temperature below the melting point of Si, while crystallization in Region III occurred after melting and solidification of Si. Whether there is an additional interaction between the Mo and Si layer enabling the crystallization at the low lattice temperatures (in Regime I) is as yet an open question.

## 6 Conclusions

We propose a single pulse fs laser crystallization process to convert a-Si to crystalline Si with and without melting using a Mo layer. Two different fluence regimes are found for nanocrystallization and polycrystallization of Si. This process offers a selective crystallization in selected regions without negatively impacting the nearby components. The crystallite size can be precisely controlled using the applied fluence. Numerical simulations confirm that nanocrystallization occurs at low temperatures at approximately half the melting point of Si, while polycrystallization of larger domains occurs at higher fluence due to melting and resolidification of Si. This study proposes ultrashort lasers as a competitive and promising tool in comparison to the direct laser crystallization of a-Si with the fs laser for rapid high speed scalable industrial manufacturing and mass production. The proposed process has a high degree of control when compared with direct laser-induced crystallization of a-Si using a similar laser.
